# Nematicidal Activity of *Burkholderia arboris* J211 Against *Meloidogyne incognita* on Tobacco

**DOI:** 10.3389/fmicb.2022.915546

**Published:** 2022-06-10

**Authors:** Renjun Zhang, Jin Ouyang, Xingyang Xu, Jie Li, Muzammal Rehman, Gang Deng, Jie Shu, Dake Zhao, Suiyun Chen, R. Z. Sayyed, Shah Fahad, Yaqiong Chen

**Affiliations:** ^1^Biocontrol Engineering Research Center of Crop Disease and Pest, Yunnan University, Kunming, China; ^2^Yunnan Engineering Research Center of Microbial Agents, Yunnan University, Kunming, China; ^3^School of Life Science, Yunnan University, Kunming, China; ^4^Kunming Branch of Yunnan Tobacco Company, Kunming, China; ^5^School of Agriculture, Yunnan University, Kunming, China; ^6^School of Ecology and Environmental Science, Yunnan University, Kunming, China; ^7^Department of Microbiology, PSGVP Mandal's S I Patil Arts, G B Patel Science and STKVS Commerce College, Shahada, India; ^8^Hainan Key Laboratory for Sustainable Utilization of Tropical Bioresource, College of Tropical Crops, Hainan University, Haikou, China; ^9^Department of Agronomy, The University of Haripur, Haripur, Pakistan

**Keywords:** nematode, *Meloidogyne incognita*, plant growth-promoting rhizosphere (PGPR), biocontrol, tobacco

## Abstract

Root-knot nematode (*Meloidogyne incognita*) is the most widespread nematode affecting Solanaceae crops. Due to the lack of effective measures to control this nematode, its management can be achieved, using biocontrol agents. This study investigated *in vitro* efficacy of the antagonistic bacterial strain J211 isolated from tobacco rhizosphere soil against *M. incognita*, and further assessed its role in controlling nematodes, both in pot and field trials. Phylogenetic analysis of the 16S rRNA gene sequence of strain J211 assigned to *Burkholderia arboris*. Culture filtrates *B. arboris* J211 exhibited anematicidal activity against the second-stage juveniles (J2s) of *M. incognita*, with a 96.6% mortality after 24 h exposure. Inoculation of J211 in tobacco roots significantly reduced the root galling caused by *M. incognita*, both in pot and field trials. Meanwhile, plant growth-promoting (PGP) traits results showed that J211 had outstanding IAA-producing activity, and the IAA production reached 66.60 mg L^−1^. In the field study, *B. arboris* J211 also promoted tobacco growth and increase flue-cured tobacco yield by 8.7–24.3%. Overall, *B. arboris* J211 as a high-yielding IAA nematicidal strain effectively controlled *M. incognita* and improved tobacco yield making it a promising alternative bionematocide.

## Introduction

Plant-parasitic nematodes, such as root-knot nematodes (RKNs), cause over $100 billion in annual crop losses, worldwide (Elling, 2013). *Meloidogyne* spp. is considered the most damaging nematodes in the world (Jones et al., 2013). A remarkable stage in the life cycle of the RKNs is the second-stage juveniles (J2s) which is the stage that has the capacity to move through soil and infect plants (Topalovi et al., 2019). RKNs severely reduce crop production by absorbing nutrients from host plants for self-reproduction (Williamson, 1999). These nematodes are a broad host range of over 2,000 plant species (Sasser, 1980), including vegetables, beans, grains, grass shrubs, fruit trees, and industrial crops (Bagheri et al., 2014). In particular, *M. incognita*, known as southern root-knot nematode, causes the most devastating root-knot diseases (Akhyani et al., 1984).

Over the past decades, several traditional strategies, including rotation, resistance breeding, and application of nematicidal agents, were used to control nematodes (Oka et al., 2012; Ralmi et al., 2016; Makunde et al., 2018). Due to monoculture or rotation with plant species that are also hosts (Nyczepir and Thomas, 2009), coupled with the difficulty of cultivating disease-resistant varieties (Sadeghi et al., 2021), RKNs control still remains extremely difficult. Control of RKNs has been mostly achieved through the use of nematicides, such as fumigants, carbamates, and organophosphates (Karavina and Mandumbu, 2012), and despite their effectiveness, nematicides are highly toxic to the natural environment and human health, that is why their use being often banned (Sharma and Sharma, 2016). During the last years, more emphasis has been given to nematode management on green and environment-friendly alternative strategies (Zhai et al., 2019).

Currently, biological control has shown an eco-friendly approach to reducing nematode damage (Cheng et al., 2017). Microorganisms are living biological agents that produce bioactive molecules and have the ability to suppress, antagonize and control nematodes in most cultivated fields (Chelinho et al., 2017). Various antagonistic bacterial and fungal species have been isolated for biocontrol of RKNs (Xiang et al., 2017; Du et al., 2020). For example, previous studies have demonstrated the biological control capability of bacterial isolates of the genera *Pseudomonas* spp. (Ali et al., 2002), *Bacillus thuringiensis* (Yu et al., 2015), *Pasteurella penetrans* (Kariuki and Dickson, 2007) and *Purpureocillium lilacinum* (Kiewnick and Sikora, 2003). Abbasi et al. (2013) used *Bacillus* spp. to reduce nematode infestation of eggplants (*Solanum melongena*). Pretreatment of tomato seeds with *Streptomyces hydrogenans* DH-16 culture supernatant reduced nematode infestation and promoted tomato seedlings' growth (Sharma et al., 2020). *Purpureocillium lilacinum* AUMC 10149 is used to control the root-knot nematode *M. incognita* infestation of tomatoes (Isaac et al., 2021). Microorganisms, the natural enemies of nematodes, inhibit nematode disease through multiple pathways: the production of toxins, antibiotics, crystal proteins, and nematicidal substances (Zhang and Mo, 2006). A cyclic dipeptide Cyclo (L-Pro-L-Leu) isolated from the metabolite of *Pseudomonas simiae* MB751 has activity against the J2s of *M. incognita* (Sun et al., 2021). *M. incognita* is synergistically controlled by the crystal proteins Cry6Aa and Cry55Aa produced by *Bacillus thuringiensis* (Peng et al., 2011). Cheng et al. (2017) revealed that 11 volatile organic compounds produced by *Paenibacillus polymyxa* KM2501-1 control *M. incognita* through multiple strategies.

Although significant progress has been made in the use of microorganisms to control RKNs in recent years, microbial root competence was considered to be a key prerequisite for successful biocontrol (Maurer et al., 2013). For this reason, new nematicidal strains with long-term survival should be isolated to control RKNs under field conditions. Plant rhizosphere soil surrounds a variety of bacteria that promote plant growth by dissolving phosphates, producing siderophores and plant growth regulators. This bacterium is called plant growth-promoting rhizobium (PGPR). PGPR is also considered a potential alternative option for controlling RKNs (Groover et al., 2020). Many studies indicated the antagonistic ability of PGPR toward RKNs, including genera *Bacillus, Serratia, Pseudomonas*, and *Burkholderia* (Gray and Smith, 2005). The rhizosphere bacterium *Pseudoxanthomonas japonensis* ZKB-2 showed strong nematostatic activity against *M. incognita* on tomatoes (*Lycopersicon esculentum*) (Hu et al., 2018). *Pseudomonas aeruginosa* and *Burkholderia gladioli* are used to control *M. incognita* on tomatoes, remarkably reducing root galls and promoting plant growth (Khanna et al., 2019). El-Aal et al. (2021) reported that mixed application of *Serratia* spp. and *Pseudomonas* spp. controlled *M. incognita* and promoted the growth of sponge gourd (*Luffa aegyptiaca*). *Bacillus cereus* Bc-cm103 isolated from cucumber (*Cucumis sativus*) rhizosphere completely killed the J2s of *M. incognita* within 12 h, and significantly reduced the infection of the nematode to cucumber root (Yin et al., 2021). Even though many microbial agents have been used to control root-knot nematodes, the activity and stability of biological agents are still affected by environmental factors, such as soil texture, moisture, and temperature.

Considering the harm of chemicals to the environment and the instability of biological agents in the current application of nematode control, it is necessary to screen an environment-friendly and sustainable method of nematode control. The objective of the present study was to evaluate the antagonistic bacterial strain J211, isolated from tobacco rhizosphere soil, for RKNs biocontrol potential on tobacco. Specifically, the purposes were to assess the nematicidal activity of strain J211 on the viability of *M. incognita in vitro* and to evaluate the efficacy of the strain as a biocontrol agent under pot and field conditions.

## Materials and Methods

### Isolation of Rhizospheric Bacteria

We followed the method described by Huang et al. (2014) with modifications to isolate the rhizospheric bacteria. Rhizosphere soil in which healthy tobacco plants grew was collected from areas infested with tobacco root-knot nematode disease. Tobacco rhizosphere soils were collected from Anning County, Yunnan Province, China (102°21′16″E, 24°56′20″N). Soil samples (1 g) were suspended in 10 mL of sterile distilled water and mixed on a table concentrator for 10 min. The soil samples were serially diluted (up to 10^−7^-fold), plated on a nutrient agar (NA) medium, and incubated at 30 ± 2°C for 2 to 3 days. Bacterial colonies growing on the plates were isolated for further study based on color and morphological characteristics.

### *Meloidogyne incognita* Population

The second-stage juveniles (J2s) of *M. incognita* were obtained from a pure population isolated in Fumin county, Yunnan province, China from tobacco (*Nicotiana tabacum* var. Hongda) roots. Eggs were extracted from roots according to Hussey and Barker (1973) and hatched in sterile water at 25–28°C for 72 h to obtain freshly hatched J2s. The collected J2s suspension was diluted with sterile water to a concentration of 5,000 per milliliter. Only freshly hatched J2s of *M. incognita* were used in the experiments.

### Toxicity Tests of Bacterial Broth Culture Filtrates

The isolated bacterial strains were screened using *in vitro* nematicidal assay, for which purpose, they were transferred to a NA medium plate. After activation on the NA plate at 30°C, purified single colonies were picked using a sterile inoculating loop and placed in a 500 ml conical flask containing 300 ml nutrient broth (NB). The isolated strains were grown in NB at 30°C for 3 days with shaking at 180 rpm and filtered through a 0.22 μm sterile filter unit to yield the culture filtrate. Three mL of bacterial broth culture filtrates were added to 35 mm Petri dishes, then adding suspension containing ~100 J2s in 20 μL sterile water. The controls were sterile NB with 100 μg mL^−1^ rifampin added to the Petri dishes. The number of dead nematodes was recorded after 12, 24, and 36 h. Viability was established using the hydroxide technique (Chen and Dickson, 2000), and nematodes without mobility are considered dead. The mortality using the Abbott (1925) formula: relative mortality (%) = [(mortality in treatment—mortality in negative control)/(1—mortality in negative control)] × 100. All treatments were conducted with five replicates and the experiment was repeated twice.

### Identification of the Bacterial Strain

The DNA of J211 was extracted by Bacterial Genomic DNA Extraction Kit (TaKaRa, Japan). The 16S rRNA gene was amplified using universal primers 27F and 1492R. The PCR products were separated on agarose gel (1%) by electrophoresis, purified with the Agarose Gel DNA Extraction Kit (TaKaRa, Japan) according to the manufacturer's instructions, and sequenced using the ABI3730xl platform (Beijing Tsingke Biological Technology Co., Ltd, China). The sequence of J211 was submitted to the NCBI GenBank database and accession numbers were obtained. The acquired 16S rRNA sequence of J211 was analyzed by using the EzBioCloud database. Other sequences of related taxa were selected from the EzBioCloud database. The Multiple Sequence Alignment was done using MEGA version 7. Phylogenetic tree of 16S rRNA gene sequences using MEGA version 7 with distance options according to the Kimura two-parameter model (Kimura, 1980) and the neighbor-joining (NJ) method, and 1,000 bootstrap replications.

### Biochemical Characterization of J211

The strain J211 was further characterized based on its biochemical characteristics as per Bergey's Manual of Systematic Bacteriology (Holt et al., 1994). Routine biochemical tests like Gram staining, spore staining, catalase activity, starch hydrolysis, gelatin hydrolysis, indole test, nitrate reduction, Methyl Red (M. R.) test, Voges-Proskauer (V. P.) test, and H_2_S production were performed for J211.

### Assessment of Plant Growth-Promoting (PGP) Traits of J211

#### IAA Production

Indole-3-Acetic Acid (IAA) production of J211 in the culture broth was determined by the colorimetric method as described by Tang and Bonner (1948) with minor modifications. J211 was grown in NB and incubated at 28 ± 2°C in an orbital incubator shaker at 180 rpm for 144 h. The cells in samples were harvested by centrifugation at 10,000 rpm for 10 min. Then, 4 ml of Salkowaski reagent (50 mL 35% perchloric acid mixed with 1 ml of 0.5% FeCl_3_) were added to 2 ml of supernatant and incubated for 30 min at room temperature in dark for the development of color, and the absorbance was measured at OD 530 nm. The concentration of IAA present in supernatant was calculated using a standard curve of IAA (Gordon and Weber, 1951).

#### Phosphate Solubilization

The strain J211 to be tested was inoculated into the Monkina inorganic phosphorus liquid medium at a dose of 1% and incubated at 28 ± 2°C and 180 rpm for 7 days. Then, 2 ml of the bacterial suspension were then centrifuged to obtain the supernatant. Dissolved phosphorus content was then quantified according to the antimony molybdenum anti-colorimetric method (Wu et al., 2012).

#### Siderophore Production

Quantitative analysis of siderophore was performed by CAS shuttle assay (Schwyn and Neilands, 1987). J211 was inoculated in a sterile siderophore inducing medium (Alexander and Zuberer, 1991) and incubated at 28 ± 2°C in an orbital incubator shaker at 180 rpm. After 7days, 1 mL supernatant of the culture was mixed with 1 mL of CAS reagent and 20 μL of Shuttle Solution (0.2 M 5-sulfosalicyclic acid). After 20 min of incubation at room temperature, absorbance was read at 630 nm. Siderophore Unit (%) was calculated using the following formula: Siderophore Unit (%) = (Ar-As)/Ar × 100, where, Ar absorbance of reference at 630 nm, As absorbance of the sample at OD 630 nm.

### Pot Experiment

Tobacco var. Hongda is extremely susceptible to root-knot nematodes, and for this reason, it was used in the assay. The potting mix (pH 6.5–7) consisted of peat and a small amount of soil amended with 0.3% (w/v) of a water-soluble fertilizer (Haoyunzhixing®, Haoshiji Chemical Industry Co., Sichuan, China). Three days before transplanting, each pot was inoculated with 3,000 J2s of *M. incognita*. Then five-leaf stage tobacco plants were transplanted into the pots (15cm diam, 13cm depth), one for each pot. J211 was inoculated in NB at 30°C for 3 days with shaking at 180 rpm, and then the fermentation broth was diluted with sterile water to adjust to 1 × 10^9^ CFU ml^−1^. The experiment was split into four treatments, including (i) untreated control with 300 mL water, (ii) 1 g of commercially available *Purpureocillium lilacinum* powder (Xinlonghui®, Xinlong Biotechnology Co., Jiangxi, China) was diluted 300-fold with water, and 300 ml of the suspension was inoculated on the roots of tobacco plants, (iii) inoculated with 300 ml culture of J211 adjusted to 1 × 10^9^ CFU ml^−1^, and (iv) 1 g of commercially available *P. lilacinum* powder was diluted 300-fold with adjusted J211 culture (1 × 10^9^ CFU ml^−1^), and 300 ml of the mixed suspension was inoculated on the roots of tobacco plants. Five replicated pots were used for each treatment. Each experiment was conducted twice, wherein plants were maintained at 25–28°C and relative humidity between 60 and 70% for 8 weeks.

Plant height was measured from five plants per treatment after 8 weeks. The root galling index (GI) was determined using a 0 to 10 rating system (Barker et al., 1986), where 0 = no galls and 10 = 90–100% of roots galled. Egg masses were stained in aqueous phloxine B and enumerated under a dissecting microscope (Dickson and Struble, 1965).

### Field Experiment

In April 2019, field trials were conducted in tobacco fields at Fumin county, Kunming, China (102°61′45″E, 25°47′34″N). The field has been in conventional tobacco products for many years and was infested with *M. incognita* (510 ± 23 nematodes per 100 g soil). The soil type was sandy clay with 38.9 g kg^−1^ of organic matter. The available nitrogen (AN), phosphorous (AP), and potassium (AK) contents were 153.6 mg kg^−1^, 68.7 mg kg^−1^, and 512 mg kg^−1^ in the field, respectively, and pH was 5.37. Potassium sulfate (150 kg ha^−1^) and N-P-K compound fertilizer (600 kg ha^−1^) were applied as basal fertilizers.

Tobacco var. Hongda was also used in the field trials of five-leaf stage plants. Five treatments were applied: (i) a 12,000-fold dilution of fluopyram (Lufta®, Bayer Crop Science, Beijing, China), (ii) a 2,000-fold dilution of fosthiazate (Sunchungtan®, Farm Hannong Co., Seoul, Korea), (iii) a 10-fold dilution of *Burkholderia arboris* J211 broth culture(J211 was inoculated in 5 L NB at 30 °C, 180 rpm for 7 days with a concentration of about 2.6 × 10^11^CFU ml^−1^), and (iv) a 300-fold dilution *P. lilacinum* (Xinlonghui®, Xinlong Biotechnology Co., Jiangxi, China), the application of (v) water was the control. 300 mL aliquot of each treatment was poured into the soil around the tobacco roots four times every 15 days. The experiment was conducted as a completely randomized block design with three replicates, each replicated plot consisted of a single row, 25 m long, with 50 tobacco plots under the same management.

To assess tobacco growth after inoculating with J211, agronomic characters, including height, stem girth, effective leaves, and leaf area, were investigated on ten random plants per plot at the topping stage (75 days after inoculation with J211). Furthermore, flue-cured tobacco yields were recorded according to the National Standard (GB 2635-1992). The disease index and control efficacy were recorded after harvest according to the National Standard (GB/T 23222−2008). Root-knot severity was rated on a standard scale from 0 to 9: 0, no symptoms; 1, less than a quarter of the roots had a few root-knots; 3, one quarter to one-third of the roots have a few root-knots; 5, one third to one-half of the roots have root-knots; 7, more than one-half of the roots have root-knots; 9, all roots are covered with root-knots. The disease index (DI) and control efficacy were calculated using the following formulas:


$$\begin{array}{l} \text{ DI = }\sum [(\text{rating }\times \text{ number of plants rated})\text{/}\\ \;(\text{total number of plants }\times \text{ the highest}\\ \;\text{rating})]\;\times \text{ 100}\\ \text{Control efficacy }(\%)\text{ = }[(\text{DI of the control - DI of the}\\ \text{ treatment})\text{/DI of the control}]\;\times \text{ 100} \end{array}$$

### Data Analysis

Data were analyzed by one-way ANOVA, and the means of the treatments were separated by Duncan's multiple-range test (*P* < 0.05) using SPSS software (version 23 for Windows; SPSS, Chicago, IL, USA).

## Results

### Identification of Bacterial Strain and Its *in vitro* Nematicidal Activity

**Figure 1:**
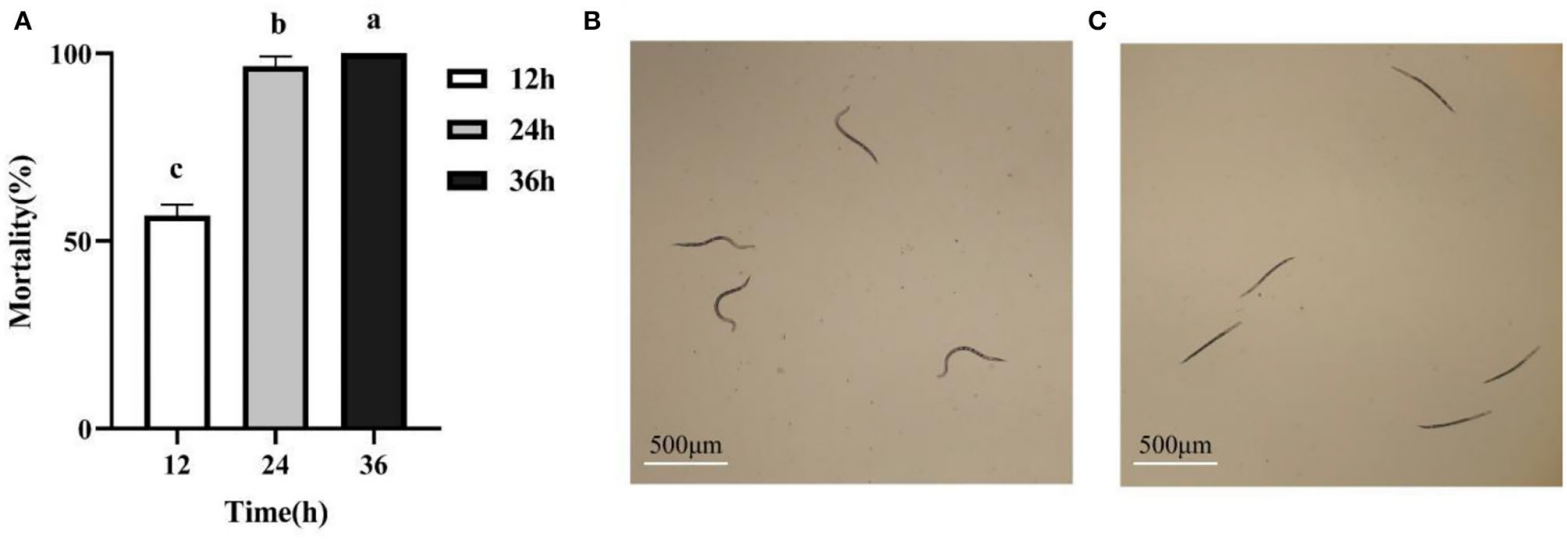
The nematocidal activity of the culture filtrates from J211 against the J2s of *M. incognita* at different times. Values are means ± standard deviation of two runs with five replicates each **(A)**. Relationships among means were analyzed by one-way ANOVA and Duncan's multiple-range test (*P* < 0.05). Means with the same letter did not differ significantly. Nematodes activity shows control, curved are vigorous **(B)**; the antagonistic stiff died and have no vitality **(C)** after 24 h. Bar: 500 μm.

**Figure 2:**
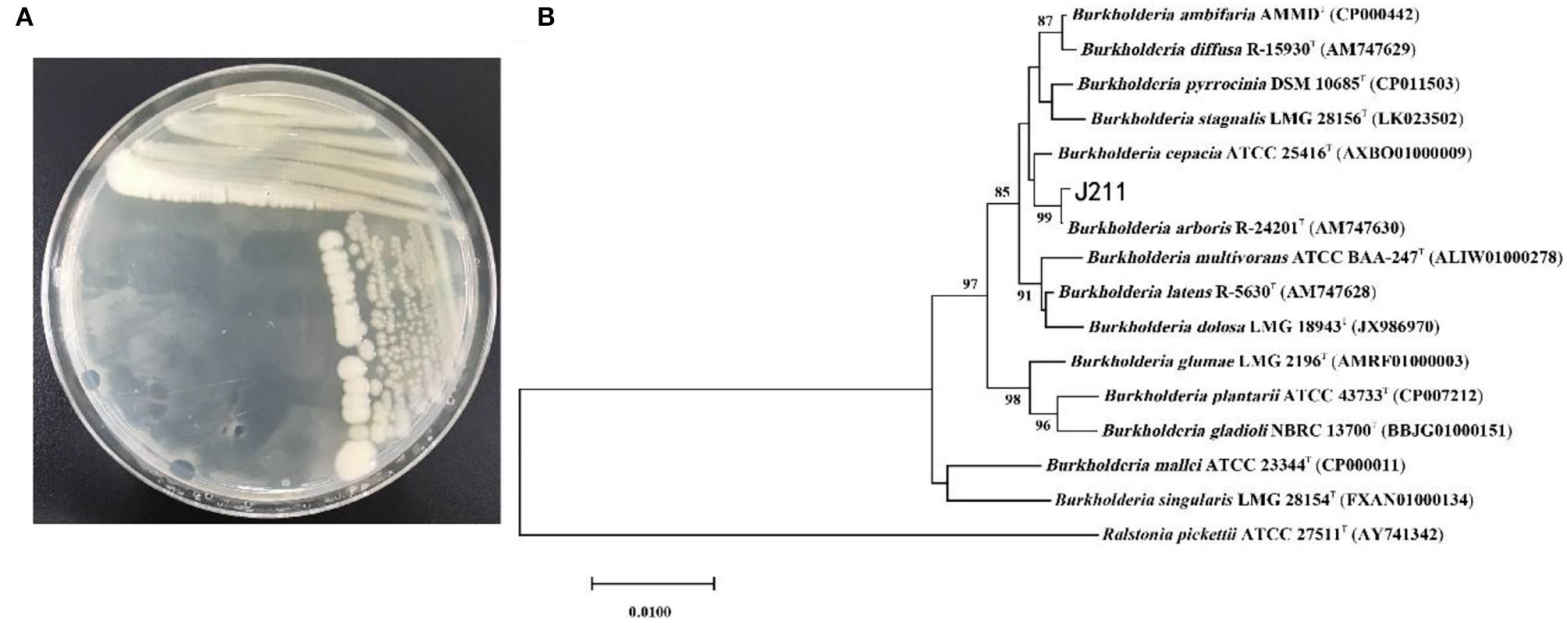
Colony morphology and phylogenetic tree of strain J211. Colony morphology of J211 on NA medium **(A)**. Phylogenetic tree constructed with J211 and other type strains of related species in *Burkholderia* based on16s rRNA gene sequences **(B)**. The bootstrap consensus tree, inferred from 1,000 replicates, was reconstructed using the neighbor-joining (NJ) method based on the general time-reversible model.

We isolated and purified 17 strains of bacteria from tobacco rhizosphere soil, and *in vitro* nematicidal assays showed that the fermentation supernatants of 4 strains had toxic effects on *M. incognita* J2s (data not shown). In particular, the isolatedJ211 showed high killing activity against *M. incognita* J2s. Testing *in vitro* displayed that the J211 culture filtrates exhibited highly nematocidal activity against J2s of *M. incognita*, with mortality close to 96.6% 24 h and 100% after 36 h (Figure 1). The colony of J211 was round, white, and non-transparent, with a smooth surface (Figure 2A). The 16S rRNA gene sequence of J211 was deposited at the NCBI GenBank database with accession MT879598. J211 was identified to the species level by using the 16S rRNA gene sequence to construct an NJ phylogenetic tree, the 16S rRNA sequence of J211 from other selected species (Figure 2B). The tree showed that J211 was located at the same branch as *Burkholderiaarboris* R-24201, with a 99% similarity to *B. arboris*.

### Biochemical Characterization and Plant Growth-Promoting (PGP) Traits of J211

The biochemical characteristics of J211 were determined according to Gram staining, spore staining, catalase test, starch hydrolysis, gelatin hydrolysis, indole test, nitrate reduction, and methyl red (M. R.), Voges-Proskauer (V. P.) and H_2_S production (Table 1). J211 had a strong ability to produce IAA, with the production amount reaching 66.60 mg L^−1^ (Table 1). Quantitative analysis showed that J211 could dissolve inorganic phosphorus, with the dissolved amount reaching 3.41 mg L^−1^ (Table 1). J211 showed a low siderophore production efficiency of only 0.19% (Table 1).

**Table 1:** Physiochemical characteristics and plant growth-promoting (PGP) properties of J211.

**Biochemical tests**				**PGP traits**	
Gram stain	-	Indole test	+	IAA production (mg L^−1^)	66.60 ± 0.31
Flagellum staining	-	Nitrare reduction	+	Phosphate solubilization (mg L^−1^)	3.41 ± 0.02
Catalase	+	R. test	+	Siderophore production (%)	0.19 ± 0.02
Starch hydrolysis	-	V. P. test	-		
Gelatin hydrolysis	+	H_2_S production	+		

*–, negative result;*

*+, positive result*.

### Effect of J211 for Control of *M. incognita* in Pot Trials

To evaluate the efficacy of J211 against *M. incognita* under potted conditions, we conducted two rounds of tobacco pot experiments. Plant height, galling index (GI), and the number of egg masses were analyzed after 8 weeks (Table 2). All nematicidal treatments, including *P. lilacinum* or J211, greatly decreased GI and egg masses compared to the untreated control. Due to the severe infestation of root-knot nematodes, the plant height of control was significantly lower than other treatments. In the treatment of inoculated with *P. lilacinum*, J211, and combined *P. lilacinum* and J211, insignificant differences were observed for height and GI, in the pot trials. Combined inoculation of *P. lilacinum* and J211 significantly reduced the number of egg masses compared to their respective inoculations alone.

**Table 2:** Effects of J211 on the growth of plants and the formation of root-knots in tobacco roots caused by *M. incognita* in the pot trial.

**Treatment**	**Height (cm)**	**Galling index (GI)**	**No. of egg masses per plant**
Control	50.4 ± 2.8b	6.1 ± 06a	167.1 ± 13.2a
*P.lilacinum*	59.9 ± 1.5a	1.7 ± 0.5b	45.3 ± 9.8b
J211	61.2 ± 2.6a	1.6 ± 0.5bc	47.2 ± 8.9b
*P.lilacinum* + J211	61.8 ± 1.6a	1.2 ± 0.4c	35.0 ± 8.4c

*Each value represents the mean ± standard deviation of one experiment with five replicates. Relationships among means were analyzed using a one-way ANOVA and Duncan's multiple-range test (P < 0.05). Means followed by the same letter within a column are not significantly different*.

### Control of Tobacco Root-Knot in Field Experiments

**Figure 3:**
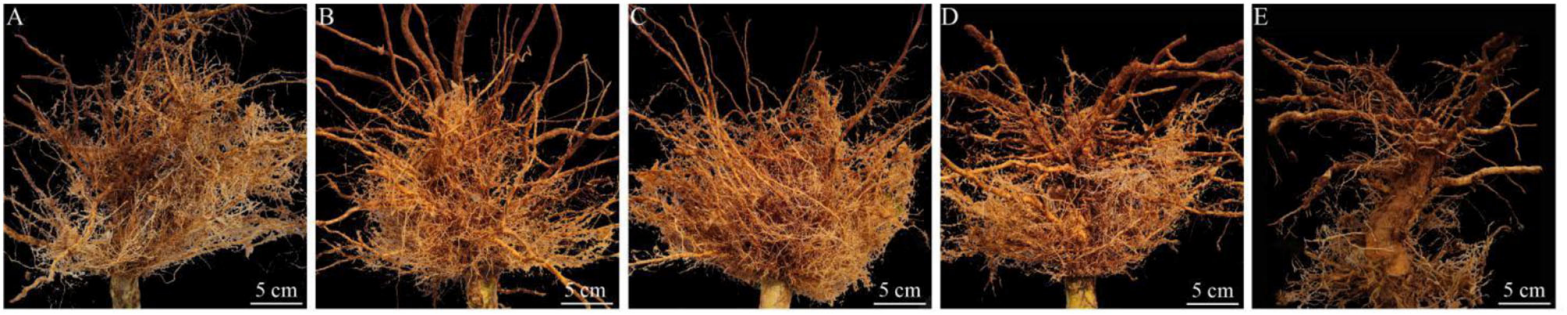
Root symptoms of tobacco with different treatments after harvest. Fluopyram **(A)**, Fosthiazate **(B)**, J211 **(C)**, *P. lilacinum* **(D)**, Control **(E)**. Bar: 5 cm.

**Figure 4:**
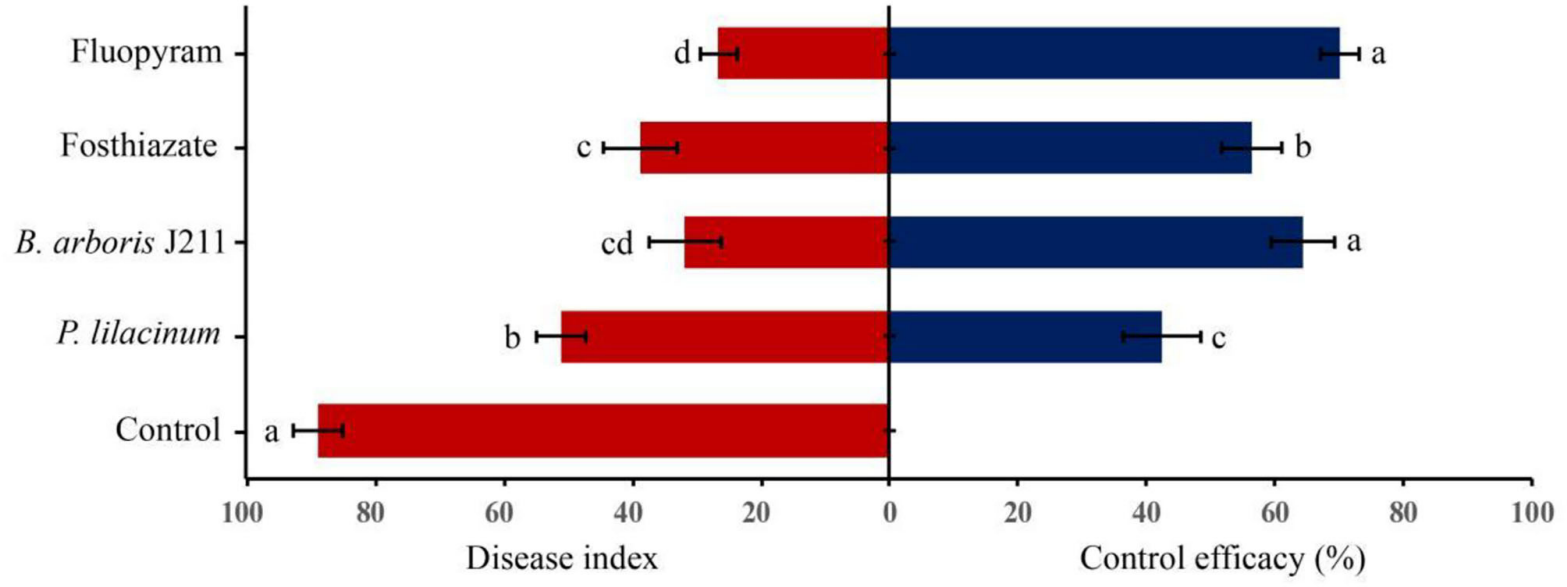
Effect of chemical and biological nematicides against tobacco RKNs in the field. Bars represent the mean ± standard deviation of three replicates (10 tobacco plants per replicate). Bars with the same letters are not significantly different (*P* > 0.05) based on Duncan's multiple-range tests.

In the field trial, the role of different treatments to nematodes was evaluated after tobacco harvest. The roots of the untreated control were severely damaged after nematode infestation, and the root knots were thick (Figure 3E). However, four other nematicidal measures significantly reduced disease symptoms at the root of tobacco (Figures 3A–D). Moreover, the broth culture of J211 at a 10-fold dilution (DI = 91.1), prominently decreased the DI of tobacco roots by 57.8 compared to untreated control (DI = 33.3) (Figure 4). No difference appeared in the tobacco DI after J211 treatment with the chemical agents fluopyram and fosthiazate. Nematicidal applications significantly increased tobacco height compared to untreated control. After chemical and biological agents treatment, caused by *M. incognita* was inhibited. The control effects were 70% (fluopyram), 56.4% (fosthiazate), 64.3% (J211), and 42.4% (*P. lilacinum*), respectively. Particularly, the application of J211 displayed a better effect on the promotion of tobacco height and stem girth, with an increase of effective leaves and leaf area, in comparison to chemical nematicides and *P.lilacinum*(Table 3). At harvest, the tobacco yield was greater in the J211 treatment than in other treatments (Table 3).

**Table 3:** Effects of different treatments on agronomic characteristics, yield, and control efficacy of *M. incognita* in tobacco field assay.

**Treatment**	**Height (cm)**	**Stem girth (cm)**	**Effective leaves**	**Leaf area (m^**2**^)**	**Yield (kg ha^**−1**^)**
Fluopyram	119.0 ± 11.4b	10.5 ± 1.4bc	18.3 ± 1.6b	0.151 ± 0.030b	2641.4 ± 156.8b
Fosthiazate	120.8 ± 20.1b	10.6 ± 1.3bc	18.5 ± 1.7b	0.151 ± 0.043b	2523.4 ± 183.5bc
J211	131.3 ± 10.3a	11.8 ± 1.1a	20.2 ± 1.1a	0.172 ± 0.025a	2872.2 ± 114.9a
*P. lilacinum*	118.6 ± 18.3b	11.1 ± 1.5b	18.5 ± 1.9b	0.153 ± 0.028b	2354.9 ± 152.1cd
Control	97.6 ± 12.1c	10.0 ± 1.6c	18.2 ± 2.9b	0.115 ± 0.037c	2311.2 ± 118.8d

*Each value represents the mean ± standard deviation of three replicates (10 tobacco plants per replicate). Means were separated using Duncan's multiple-range test (P < 0.05). Means with the same letter are not significantly different*.

## Discussion

Root-knot nematodes are severe threats to world agriculture. The application of bacteria especially PGPR, and biological control of RKNs have drawn increasing attention (Siddiqui and Futai, 2009; Xiang et al., 2017). *In vitro* tests from this study indicated J211 a high mortality of *M. incognita* J2s. Phylogenetic analysis assigned the strain J211 to *B. arboris*. To our knowledge, this is the first report of antagonistic activity shown by a strain of *B. arboris* against *M. incognita*.

Both the pot and field trials demonstrated the potential of *B. arboris* J211 as a biological nematicide for the management of RKNs in tobacco cultivars. The use of biocontrol bacteria for the management of nematodes is difficult due to inefficient bacterial colonization (Liu et al., 2020). It is stated that PGPR has a strong ability to survive in and colonize rhizosphere soil (Saharan and Nehra, 2011). High control efficacy was obtained by drenching the soil with rhizosphere isolates bacteriaJ211to likely ensure colonization, and drastically reduced RKNs incidence. In pot experiments, biocontrol treatments, including inoculation with J211, *P. lilacinum*, and mixed inoculation of J211 and *P. lilacinum*, significantly reduced nematode-induced root damage compared to controls. The results of the pot experiment showed that the three biocontrol treatments had no significant difference in root galling index (GI) caused by nematodes. To evaluate the efficacy of J211 in controlling nematodes under field conditions, we compared the effects of chemical and biocontrol fungi treatments. The application of J211 reduced the root damage with a nematode control efficacy similar to fluopyram or even greater than that of fosthiazate and *P. lilacinum*in in the field experiment. *P. lilacinum*, an egg-parasitic fungus with the ability to infect and destroy nematode eggs, has become a commercial biological agent for nematode control (Mendoza et al., 2004; Baidoo et al., 2017). Indeed, the stability of biologics is affected by a variety of environmental factors. *P. lilacinum* was significantly less effective than J211 in controlling nematodes under field conditions. The possible explanation is that J211 isolated from tobacco rhizosphere soil is more suitable to colonize tobacco rhizosphere soil to play its role. The present study revealed the biocontrol potential of J211 in controlling *M. incognita*. PGPR mediated plant resistance toward RKNs is provided by the production of various antagonistic compounds, lytic enzymes, toxins, and antibiotics that inhibit the nematode proliferation or directly kill them (Cetintas et al., 2018). In this study, we observed that the effect of J2s completely killed by J211 fermentation filtrate within 36 h may be somewhat similar to that reported by Köthe et al. (2003) that *Burkholderia cepacia* culture filtrate containing extracellular toxin killed *Caenorhabditis elegans* within 24 h. However, the specific mechanism of action of J211 against *M. incognita* is not understood.

Furthermore, we measured the PGP traits of J211 and found that the ability to dissolve phosphate and produce siderophore was not outstanding, but noted that J211 was a high-yielding strain of IAA with a yield of up to 66.6 mg L^−1^. Tobacco inoculated with J211 gave rise to an 8.7–24.3% flue-cured tobacco yield increase in this study. On the one hand, J211 reduced nematode damage to tobacco roots, and on the other hand, it may be related to J211, which produces IAA activity, and promoted root growth. J211 had a similar nematode control effect as the chemical agent fluopyram, but J211 promoted the growth of lateral roots of tobacco more obviously (Figure 3C). PGPR has been described as being able to fortify plant development by regulating plant hormones, such as auxins, gibberellins, cytokinin, and ethylene (Tahir et al., 2017). Auxin is a phytohormone that regulates most plant processes. Though endogenous auxin synthesis occurs in plants, it also depends on the external supply of auxin which can be fulfilled by PGPR isolates that occur in the rhizosphere of the plant (Patten and Glick, 2002). Islam et al. (2015) reported that cucumber rhizosphere isolates, including *Pseudomonas stutzeri, Bacillus subtilis, Stenotrophomonas maltophilia*, and *Bacillus amyloliquefaciens*, produced IAA levels as high as 26.78−51.28 mg L^−1^. Treatment of these PGPR strains significantly promoted cucumber growth. In addition, the *B. arboris* CSRS12 isolated by Singh et al. (2020) promoted mung bean (*Vigna radiata*) lateral root growth by solubilizing phosphate and siderophores. This is different from the underlying mechanism that *B. arboris* J211 promotes tobacco plant growth through high-yield IAA observed in this study.

Unlike chemical nematicides, bacterial agents probably create no harm to humans, animals, and the environment (Rahman et al., 2018; Vurukonda et al., 2018). J211 can be safely and continuously used during crop cultivation to control nematodes and has excellent IAA producing activity, which significantly promoted the growth of tobacco plants and improved the yield of flue-cured tobacco. PGPR produces bioactive substances in the rhizosphere to resist pathogens and promote plant growth. PGPR have a distinct advantage over the obligate nematode parasite *Pasteuria penetrans* as potential biocontrol agents because they can establish in the rhizosphere independently of the nematode population. In conclusion, the utilization of PGPR is treated as an environmentally sound and promising method for the management of RKNs and increases crop yields through both direct and indirect mechanisms (Lugtenberg and Kamilova, 2009; Vejan et al., 2016).

## Conclusion

In this study, the strain of *Burkholderia arboris*J211 was isolated from the rhizosphere soil of tobacco plants. The nematicidal assay test demonstrated the high nematicidal activity of J211 fermentation metabolites within 24 h. Inoculation with J211 significantly reduced infestation of tobacco roots by *M. incognita* in pot and field experiments. Moreover, J211 is a high-yielding strain of IAA, and the yield of tobacco plants treated with J211 was significantly increased. It is, however, necessary to find how J211 acts against RKNs, and the active substance involved, to develop a potential bionematicide from this isolate.

## Data Availability Statement

The raw data supporting the conclusions of this article will be made available by the authors, without undue reservation.

## Author Contributions

YC and SC designed the experiments. RZ and JO designed and carried out the experiments and wrote the manuscript. XX, JL, JS, and DZ participated and analyzed the data from the experiments. MR, GD, SF, and RS reviewed the manuscript. All authors contributed to the article and approved the submitted version.

## Funding

This work was financially supported by the Key Special Project of Science and Technology of the Yunnan Branch of China National Tobacco Corporation (Contract No. 2021530000241031).

## Conflict of Interest

JO, XX, and JL are the employers of Yunnan Tobacco Company. The remaining authors declare that this research was conducted in the absence of any commercial or financial relationships that could be construed as a potential conflict of interest.

## Publisher's Note

All claims expressed in this article are solely those of the authors and do not necessarily represent those of their affiliated organizations, or those of the publisher, the editors and the reviewers. Any product that may be evaluated in this article, or claim that may be made by its manufacturer, is not guaranteed or endorsed by the publisher.
